# Free-Energy Simulations Support a Lipophilic Binding Route for Melatonin Receptors

**DOI:** 10.1021/acs.jcim.1c01183

**Published:** 2021-12-21

**Authors:** Gian Marco Elisi, Laura Scalvini, Alessio Lodola, Marco Mor, Silvia Rivara

**Affiliations:** † Dipartimento di Scienze degli Alimenti e del Farmaco, Università degli Studi di Parma, Parco Area delle Scienze 27/A, I-43124 Parma, Italy; ‡ Microbiome Research Hub, University of Parma, I-43124 Parma, Italy

## Abstract

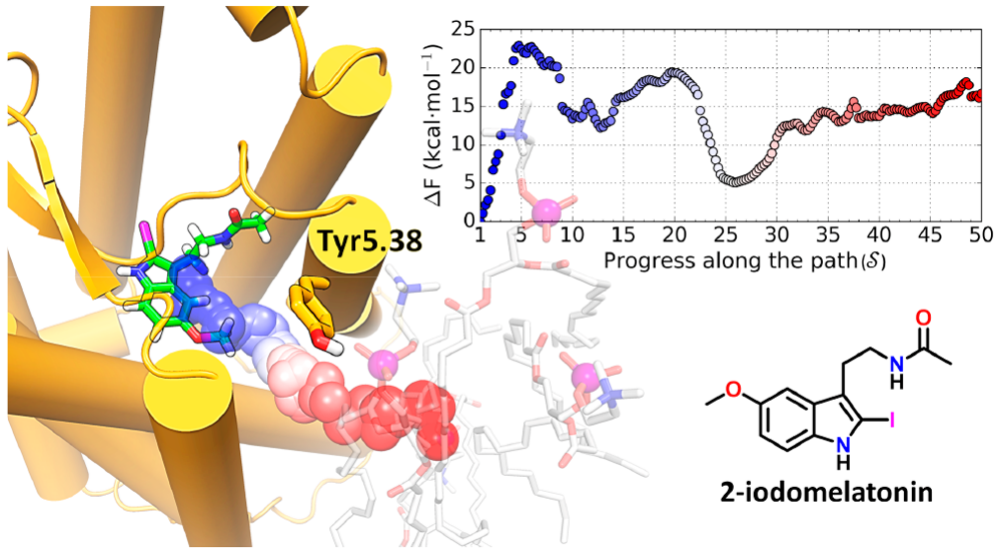

The effects of the neurohormone melatonin are mediated by the activation of the GPCRs MT_1_ and MT_2_ in a variety of tissues. Crystal structures suggest ligand access to the orthosteric binding site of MT_1_ and MT_2_ receptors through a lateral channel between transmembrane (TM) helices IV and V. We investigated the feasibility of this lipophilic entry route for 2-iodomelatonin, a nonselective agonist with a slower dissociation rate from the MT_2_ receptor, applying enhanced sampling simulations and free-energy calculations. 2-Iodomelatonin unbinding was investigated with steered molecular dynamics simulations which revealed different trajectories passing through the gap between TM helices IV and V for both receptors. For one of these unbinding trajectories from the MT_1_ receptor, an umbrella-sampling protocol with path-collective variables provided a calculated energy barrier consistent with the experimental dissociation rate. The side-chain flexibility of Tyr5.38 was significantly different in the two receptor subtypes, as assessed by metadynamics simulations, and during ligand unbinding it frequently assumes an open conformation in the MT_1_ but not in the MT_2_ receptor, favoring 2-iodomelatonin egress. Taken together, our simulations are consistent with the possibility that the gap between TM IV and V is a way of connecting the orthosteric binding site and the membrane core for lipophilic melatonin receptor ligands. Our simulations also suggest that the open state of Tyr5.38 generates a small pocket on the surface of MT_1_ receptor, which could participate in the recognition of MT_1_-selective ligands and may be exploited in the design of new selective compounds.

## Introduction

**Figure 1:**
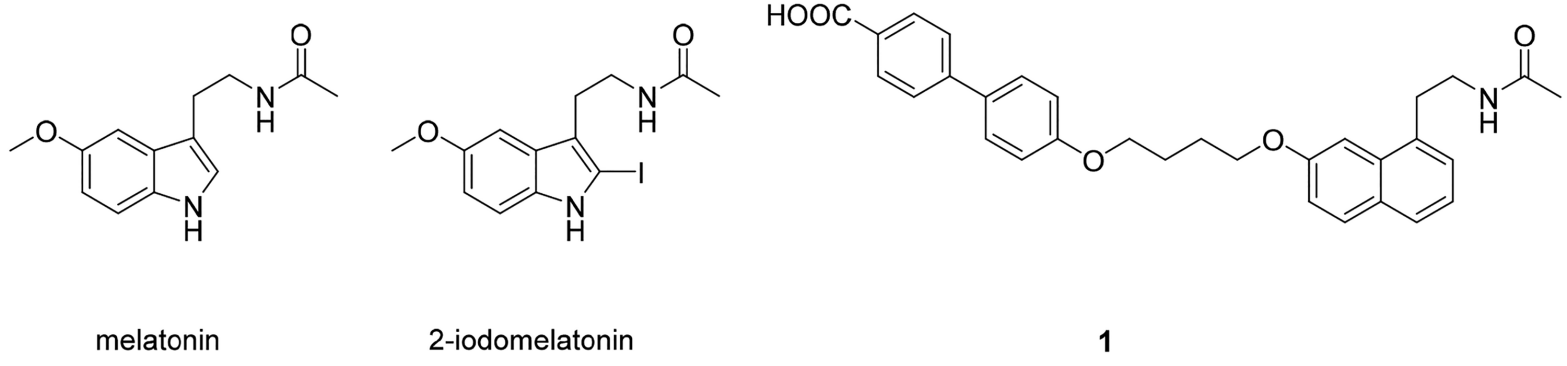
Chemical structures of melatonin, 2-iodomelatonin, and the MT_1_-selective ligand **1** (*N*-[2-(7-{4-(4′-carboxybiphenyl-4-yloxy)butoxy}naphthalen-1-yl)ethyl]acetamide).^10^

Melatonin (*N*-acetyl-5-methoxytryptamine, Figure 1, compound **1**) is a neurohormone mainly synthesized by endocrine cells situated in the pineal gland following the circadian rhythm, with elevated levels at night. Melatonin is described as a pleiotropic molecule with a multiplicity of effects^1^ and a variety of cellular targets.^2^ In mammals, modulation of several physiological and neuroendocrine functions occurs through activation of the two class A G-protein-coupled receptors (GPCRs) MT_1_ and MT_2_, for which melatonin shows subnanomolar binding affinity.^3,4^ Activation of the two receptors is involved in the entrainment of circadian and seasonal rhythms and in regulation of the sleep-wake cycle,^5^ as well as in a multitude of other physiological functions, comprising regulation of body temperature and hormone secretion, homeostasis of glucose secretion and of the cardiovascular system, and pain perception. Therefore, targeting of these receptors is of clinical interest for the treatment of pathologies affecting both the central nervous system^6^ and peripheral sites.^7^ Several melatonin receptor ligands have been synthesized, with distinct levels of intrinsic activity and receptor subtype selectivity,^8,9^ and MT_1_/MT_2_ nonselective agonists have been approved for the treatment of insomnia, circadian rhythm disorders, and major depression.

One of the most relevant observations coming from the three-dimensional structures of melatonin receptors^11−13^ is related to the entrance route of ligands to the binding site. In fact, crystal structures of class A GPCRs for polar ligands (e.g., aminergic neurotransmitters, adenosine) highlight access to the orthosteric binding site, within the 7-transmembrane (TM) bundle, from the aqueous phase in contact with the extracellular side.^14^ On the contrary, for lipophilic or amphiphilic ligands, entrance to the binding site through the membrane bilayer has been proposed.^15,16^ In sphingosine-1-phosphate, free fatty acid receptor 1, and cannabinoid receptors crystal structures, ligands can gain access to the binding site through gaps between the TM helices.^17−19^ Given its lipophilic character, melatonin easily permeates the biological membranes,^20,21^ whose interfacial region could, in principle, be able to concentrate the ligand prior to the binding process.^22,23^

**Figure 2:**
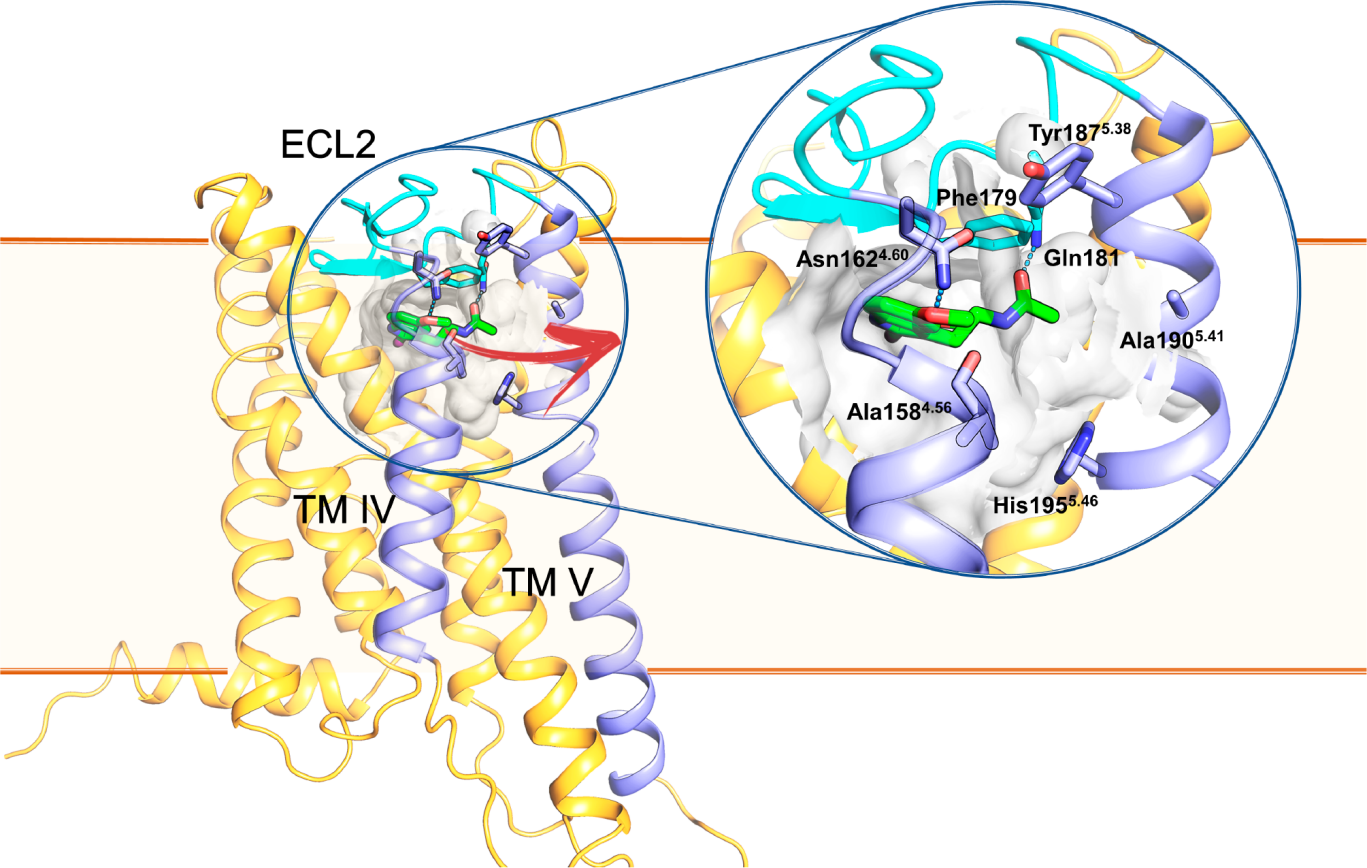
Structure of the MT_1_ receptor crystal structure (PDB id 6ME4) in complex with 2-iodomelatonin (green carbon sticks) is represented embedded in a stylized membrane bilayer with approximate boundaries represented as orange lines. The binding site (depicted as a white surface around the ligand) is enclosed in the extracellular side of the TM domain and shielded from the extracellular aqueous environment by ECL2 (light blue cartoon). ECL2 participates in the shaping of the binding site, contacting the ligand through a hydrogen bond between Gln181 and the ethylamide carbonyl group and through hydrophobic contacts between Phe179 and the indole ring. Additionally, the methoxy group interacts with Asn162^4.60^. In this work, the unbinding of 2-iodomelatonin through an opening between TM helices IV and V (purple ribbons) has been simulated (red arrow). Tyr187^5.38^, located at the boundary of the TM channel, is found in an “open” conformation with the side chain pointing toward the membrane. Mutants of Ala158^4.56^ and Ala190^5.41^ having bulkier side chains led to a decreased functional activity, likely due to the reduced width of the TM channel.^11^

The existence of a lateral channel (Figure 2) that could serve for ligand entry from the lipid bilayer has been described for melatonin MT_1_ and MT_2_ receptors.^11,12^ In crystal structures, the extracellular loop 2 (ECL2) adopts a β-hairpin structure which seals off the top of the receptor and could prevent ligand entrance from the extracellular side. Ligand access is proposed to occur through a channel between TM helices IV and V, lined by hydrophobic residues, which opens toward the outer lipid layer of the membrane. The existence of this lateral ligand access is supported by mutagenesis studies at the MT_1_ receptor in which replacement of Ala158^4.56^ or Ala190^5.41^ (Figure 2, Ballesteros-Weinstein numbering is adopted throughout the text),^24^ bordering the entrance channel, with bulkier residues causes decrease or loss of functional activity for agonist compounds.^11^

In crystal structures of MT_1_ and MT_2_ receptors captured in their inactive conformations, melatonin and other nonselective agonists showed similar binding poses and interactions.^11,12^ The amide side chain of ligands undertakes a hydrogen bond with an asparagine in ECL2 (Asn181/194 in MT_1_ and MT_2_, respectively), and the methoxy oxygen is bound to Gln162/175^4.60^. ECL2, which participates in the shaping of the binding site, further contacts the aromatic nucleus of the ligand through a phenylalanine residue (Phe179/192^ECL2^) via hydrophobic contacts. The binding site residues and their spatial arrangements are highly conserved among the two subtypes, with one interesting difference in the orientation of Tyr187/200^5.38^, on top of the channel between TM helices IV and V. In the MT_1_ crystal structure, the side chain of Tyr5.38 assumes an “open” state and points toward the lipid interface (Figure 2), while in the MT_2_ receptor, it interacts with Ala158^4.56^ in a “closed” state, constricting the channel. Despite the high similarity in the MT_1_ and MT_2_ binding sites, kinetic experiments revealed longer residence times for [^3^H]-melatonin and 2-[^125^I]-iodomelatonin at the MT_2_ receptor.^12,25^ In particular, a *k*_off_ of 2.41·10^–4^ s^–1^ is reported for 2-iodomelatonin for the MT_1_ receptor at room temperature, while no dissociation could be observed from the MT_2_ receptor for 4 h.

In this work, we used free-energy simulations to evaluate the viability of a lipophilic route for ligand entrance/exit from the orthosteric binding site of the MT_1_ receptor. Applying enhanced-sampling simulations, 2-iodomelatonin (Figure 1) was forced to exit from the receptor through the lateral channel. Ligand unbinding was thus simulated with an energy dissociation barrier in agreement with experimental *k*_off_ at the MT_1_ receptor,^25^ supporting the possibility that dissociation can occur toward the membrane, crossing the space between TM helices IV and V. Moreover, analysis of MT_1_ and MT_2_ structures and behavior during molecular dynamics simulations of ligand unbinding proposed a role for Tyr5.38 flexibility as the structural determinant at the basis of the slower ligand dissociation from the MT_2_ receptor compared to the MT_1_ receptor and suggested the possibility to exploit this difference for the design of selective ligands.

## Methods

### Protein Preparation

Crystal structures of the MT_1_ and MT_2_ receptors in complex with 2-iodomelatonin (PDB id 6ME4)^11^ and 2-phenylmelatonin (PDB code 6ME6),^12^ respectively, were prepared for molecular modeling studies according to an already published procedure.^26^ In the MT_2_-2-phenylmelatonin complex, the phenyl ring was replaced with an iodine atom to obtain 2-iodomelatonin. Molecules belonging to buffers used for crystallization were removed. Residues encompassing the *N*-terminal sequence in the MT_2_ receptor (linked to the thermostabilized apocytochrome b562RIL) were removed, leaving Pro36 as the first residue. Intracellular loop 3 (ICL3, Gln219-Pro227 in MT_1_ and Arg232-Leu240 in MT_2_ receptors) and missing side chains were added with Modeller 9.21.^27^ One hundred models were generated for each structure, leaving residues adjacent to the reconstructed loop flexible to allow proper geometries (Arg218 and Lys228 for the MT_1_ receptor, Arg231 and Cys241 for the MT_2_ receptor), while the rest of the protein was kept frozen during the optimization of the spatial restraints. The models were ranked according to the built-in molecular probability density function (molpdf),^28,29^ and those with the lowest molpdf values were further processed.

The thermostabilizing mutations in the crystal structures were reverted to the wild-type residues with Maestro 11.6^30^ graphical interface, and the structures were processed by adding hydrogen atoms and termini caps with the Protein Preparation Wizard tool of the Maestro Suite.^31,32^ ICL3 residues and the side chains of the modified amino acids were submitted to an energy minimization with the OPLS3e force field^33^ implemented in MacroModel 12.0^34^ in an implicit water solvation model,^35^ using the Polak-Ribière conjugate gradient method^36^ to a convergence threshold of 0.05 kJ·mol^–1^·Å^–1^.

The orientation of thiol and hydroxyl groups and the conformation of asparagine, glutamine, and histidine residues were sampled to optimize the overall hydrogen bonding network. Basic and acidic amino acids were modeled in their charged protonation state, while histidine residues were modeled in their neutral form. The tautomeric state of histidine residues was chosen coherently with the optimization of the hydrogen bonding network. His195/208^5.46^, located on the border of the TM opening,^11^ was modeled in its distal tautomeric state.

The final structures were energy-minimized through a first minimization run allowing relaxation of hydrogen atoms, followed by a second minimization run with heavy atom positions restrained to an RMSD value of 0.3 Å, as implemented in the Protein Preparation Wizard workflow.

### System Building and Parametrization

The protein–ligand complexes were embedded in a 1-palmitoyl-2-oleyl-*sn*-glycerol-3-phosphocholine (POPC) bilayer consisting of 150 residues by using the membrane model builder of the Charmm-GUI server^37^ according to the preorientation provided by the OPM database^38^ and then solvated in a TIP3P water environment^39^ of about 81 × 81 × 107 Å.

The systems were then parametrized with the t-leap module:^40^ the protein was parametrized by applying the ff14SB Amber Force Field^41^ and the ligand according to the general Amber force field (GAFF).^42^ The parameters for the POPC bilayer belong to the Lipid17 Force Field set. Neutrality for each system was obtained by adding 16 and 10 chloride ions to the MT_1_ and MT_2_ boxes, respectively, according to ion parameters from ref (43). Partial atomic charges of 2-iodomelatonin were computed with Jaguar 10.0^44,45^ at the Hartree–Fock level in the gas phase with the LACV3P* basis set through a RESP procedure,^46^ while the 6-31G* basis set was employed for compound **1**.

The complex of the MT_1_ receptor with compound **1** was obtained via docking calculations described in Paragraph S1 and Figure S1.

### Molecular Dynamics Simulations

Molecular dynamics (MD) simulations were performed using Gromacs 2019.2.^47^ Biased simulations were conducted by patching the MD code with Plumed 2.5.4.^48^ Long-range electrostatics were computed with the Particle Mesh Ewald summation^49^ with a Fourier scheme adopting fourth order interpolation and 1.6 Å grid spacing, while short-range and Lennard-Jones interactions were computed with a 10 Å cutoff. Bond lengths of hydrogens bound to heavy atoms were restrained to their equilibrium values with the LINCS algorithm^50^ to consent to the use of an integration time step of 2 fs. Details about systems’ equilibration before enhanced sampling simulations are provided in Paragraph S2. MD simulations were performed in canonical ensemble at 298 K, controlled under the Nosé–Hoover thermostat^51,52^ with a coupling constant of 0.5 ps. An isotropic force constant of 0.1 kcal·mol^–1^·Å^–2^ was applied to a set of 89 backbone carbons restrained to their position in the crystal structures to maintain the overall geometry of the TM bundle, while allowing movements of the upper portion of TM helices during ligand unbinding. The list of restrained atoms is reported in Paragraph S3.

### Steered MD Simulations

Steered MD (SMD) simulations were used to compute the work (*W*) required for the unbinding of 2-iodomelatonin from the MT_1_ and MT_2_ receptors by modifying the Hamiltonian of the system along chosen degrees of freedom called collective variables (*CV*s) through the application of a moving harmonic restraint^53^
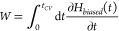
1where  with *X* as the microscopic coordinates of the system and *H*_*MD*_ as the unbiased Hamiltonian.

**Figure 3:**
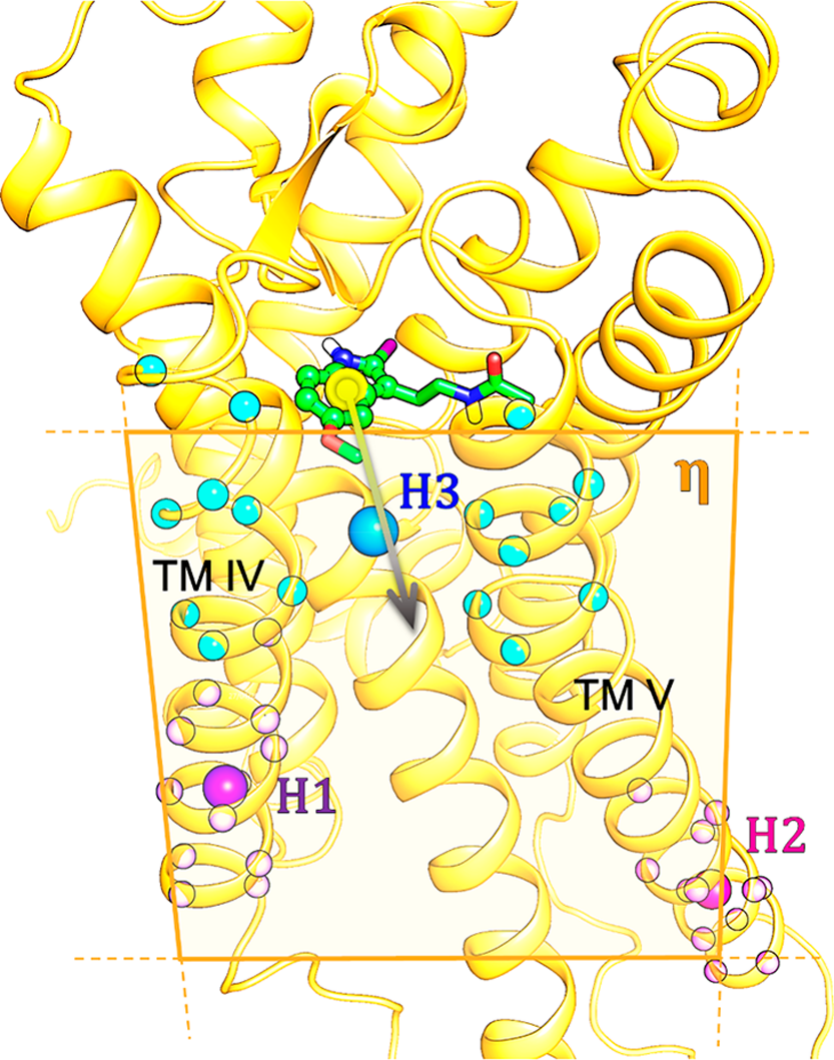
Definition of the unbinding *CV*: *d*(*COM*_*indole*_, η) for 2-iodomelatonin (green sticks, with indole heavy atoms represented as spheres) unbinding from the MT_1_ receptor in SMD simulations. The distance of the center of mass of the indole heavy atoms (yellow sphere) is calculated from the η plane (orange plane), defined through three centers of mass: H1 (purple sphere) is the center of mass of the restrained alpha carbons on TM IV (small light purple spheres), H2 (pink sphere) is the center of mass of the restrained alpha carbons on TM V (small light pink spheres), and H3 (cyan sphere) is the center of mass for unrestrained alpha carbons belonging to the extracellular sides of TM helices IV and V (small light cyan spheres). 2-Iodomelatonin is forced to move by restraining the distance of the center of mass of the indole ring from the η plane at different values. The unbinding *CV* of 2-iodomelatonin from the MT_2_ receptor was defined in the same way.

Twenty simulations were performed for each receptor complex to evaluate the unbinding process through the opening between TM helices IV and V. A *CV* was designed to describe the distance of the center of mass (*COM*) of the indole heavy atoms of 2-iodomelatonin from a η plane defined by the three centers of mass obtained from selected alpha carbons of TM helices IV and V (H1, H2, and H3 in Figure 3). The distance was calculated according to eq 2
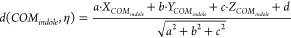
2where *a*, *b*, *c*, and *d* are the coefficients of the implicit equation of the η plane (details about plane definition are reported in Paragraph S4).

During the first 100 ps of simulation, the moving harmonic restraint was linearly increased to 50 kcal·mol^–1^·Å^–2^ on *d*(*COM*_*indole*_, η) = −8 Å, which is the closest unitary value of the *CV* to the equilibrium position of the center of mass of the indole observed during the last 100 ns of MD simulation of the equilibration step. Then, the harmonic restraint was moved from *d*(*COM*_*indole*_, η) = −8 to 12 Å in 30.0 ns (a rate corresponding to 0.66 × 10^–3^ Å·ps^–1^), in stiff spring approximation regime.^54^

### Path-like Collective Variables-Umbrella Sampling (PCV-US) Simulations

**Figure 4:**
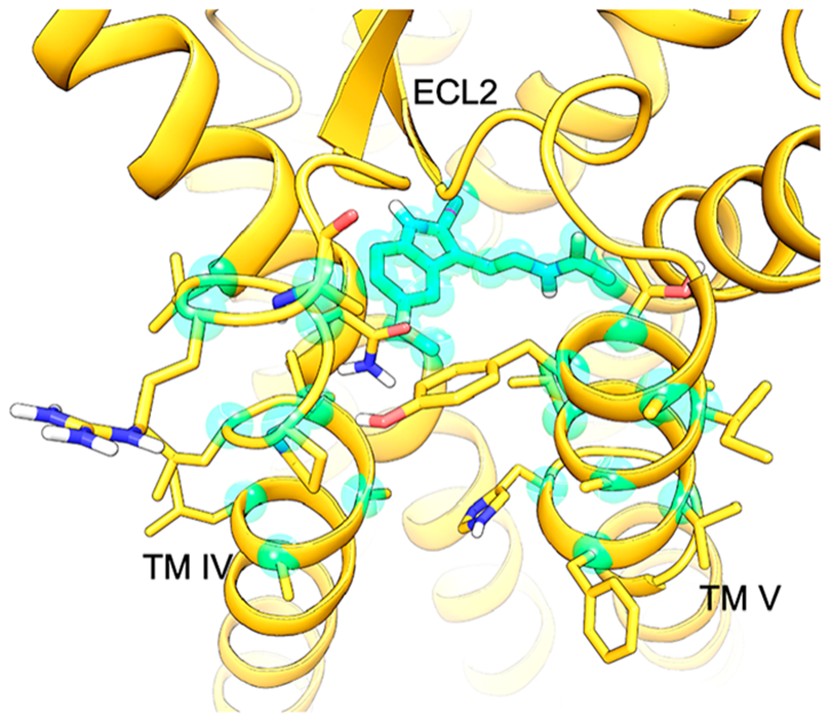
Coordinates of ligand heavy atoms and selected alpha carbons (Leu156^4.54^-Ala165^4.63^, Ala186^5.37^-His195^5.46^), belonging to TM helices IV and V of the MT_1_ receptor, are used to define reference configurations for the PCV function. These atoms are colored cyan in the picture.

One of the unbinding trajectories of 2-iodomelatonin from the MT_1_ receptor obtained from SMD simulations (run #14) was used to define path *CV*s (PCVs)^55^ for the evaluation of the unbinding free-energy profile of 2-iodomelatonin. Fifty reference configurations were extracted, based on the position of the ligand heavy atoms and selected alpha carbons of TM helices IV and V and ECL2 (Figure 4). The PCV technique^55^ employs two high-dimensional *CV*s ( and , defining the position of atoms included in the path with respect to a set of reference configurations (), with  describing the progress along the path (eq 3) and  defining the distance from the same path (eq 4)
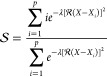
3
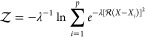
4where *i* is a discrete index ranging from 1 to *P* = 50, [(*X*–*X*_*i*_)] is the root-mean-square displacement between the instantaneous configuration of the atoms included in the path and the state *i*^*th*^ of the frameset, and λ is a smoothing parameter. The metric adopted for PCVs was the root-mean-square deviation, calculated after the alignment of selected atoms (Figure 4) on their reference configurations with the optimal alignment matrix, calculated through the Kearsley algorithm.^56^

The 50 reference configurations were obtained through consecutive pulling simulations to optimize the initial guess provided by the SMD simulation (details are reported in Paragraph S5). Given the similar work profile obtained for the last three pulling simulations (Figure S2), the fourth run was used to generate the 50 reference configurations for PCV-US simulations. Before PCV-US simulations, a restraint on the *CV* was placed at = 0.25 Å^2^ applying a force constant linearly increasing from 5 to 1,000 kcal·mol^–1^·Å^–4^ in a 1 ns simulation, while the force constants on  unitary values were linearly reduced from 50 kcal·mol^–1^ to 5 kcal·mol^–1^.

PCV-US simulations^57−59^ were conducted to estimate a monodimensional free energy profile^60^ over the *CV*, using the Weighted Histogram Analysis Method (WHAM)^61^ to reconstruct the potential of mean force (PMF). The bias potential in the form of a harmonic restraint of 5 kcal·mol^–1^ was kept for 50 ns for each of the  unitary values corresponding to a reference configuration represented in the final frameset, with a smoothing parameter λ = 6.936 Å^–2^. To ensure the overlap of neighboring simulations with a high free-energy derivative, windows with  = 2, 3, 4, 5, and 8 were simulated twice, once with a spring constant of 5 kcal·mol^–1^, as the other US windows, and once with a spring constant of 10 kcal·mol^–1^.

Different diagnostic criteria^62^ were used to assess the reliability of the obtained PMF: (i) time series of the PMF (Paragraph S6, Figure S3), (ii) the overlap of the biased probability distributions in the neighboring US windows (Figure S4), and (iii) the qualitative agreement of the *a priori* probabilities for the neighboring US windows assessed from the dissection of the global PMF into the free-energy curves of the individual simulations (Figure S5).

### Well-Tempered Metadynamics (MetaD) Simulations

The conformational space of Tyr187/200^5.38^ was sampled performing well-tempered MetaD simulations.^63^ A history-dependent bias acting on the side-chain dihedral angles selected as *CV*s was added in the form of Gaussian potentials *V* of width σ and height *h* deposited at a constant time interval τ, each one being centered on the value of the *CV*s assumed at the moment of the deposition. At the simulation time *t*, after *j* depositions (with *j*τ ≤ *t*), the total potential deposited in the point characterized by the vector ***cv*** in the *n*-dimensional space of the collective variables *CV*_*i*_ is
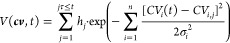
5

With the well-tempered implementation, the height *h* in eq 5 is rescaled from the initial height *h*_0_ over time as a function of the previously deposited potential and visited coordinates (eq 6)
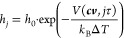
6by setting Δ*T* through a bias factor . Parameters adopted for simulations are reported in Table S1.

Free-energy differences between energy basins were estimated by averaging monodimensional MetaD simulations performed using Tyr5.38 χ_1_ dihedral angle as a single *CV*. The free-energy profiles were obtained by averaging the probability distributions associated with the free-energy surfaces of 20 independent well-tempered MetaD simulations, started with different seeds for the distribution of initial random velocities. For the *i*^*th*^ simulation, the normalized probability *P*_*ij*_ for the system to be found in the *j*^*th*^ bin, within a small interval of the *CV*, was obtained from the corresponding free-energy profile via
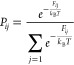
7where *F*_*ij*_ is the free energy calculated by the MetaD protocol for the center of the *j*^*th*^ bin, and the sum at the denominator was extended to the whole range of the dihedral angle used as the *CV*, to satisfy the normalization condition Σ_*j*=1_*P*_*ij*_ = 1. The bins were 3°-large, to give a good approximation of the continuous free-energy function calculated by MetaD.

The average free energy, with its uncertainty, computed as the SEM of the different probability-averaged free-energy profiles, is described by eq 8
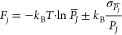
8where *P*_*j*_ is the average of the normalized probability distributions of all the simulations.

The simulations were considered finished after the potential deposition did not reach one-tenth of the initial height (i.e., 0.02 kcal·mol^–1^) for at least 10,000 consecutive depositions (30.0 ns), similar to what was reported in ref (64). Simulations lasted on average about 164 ± 28 ns and 158 ± 36 ns for the MT_1_ and the MT_2_ receptors, respectively.

Additionally, bidimensional MetaD simulations, based on Tyr5.38 χ_1_ and χ_2_ dihedral angles as the *CV*s, were performed to compare the results with those of the monodimensional simulations (Figures S6 and S7).

## Results and Discussion

### Evaluation of the Free-Energy Profile of 2-Iodomelatonin Unbinding

**Figure 5:**
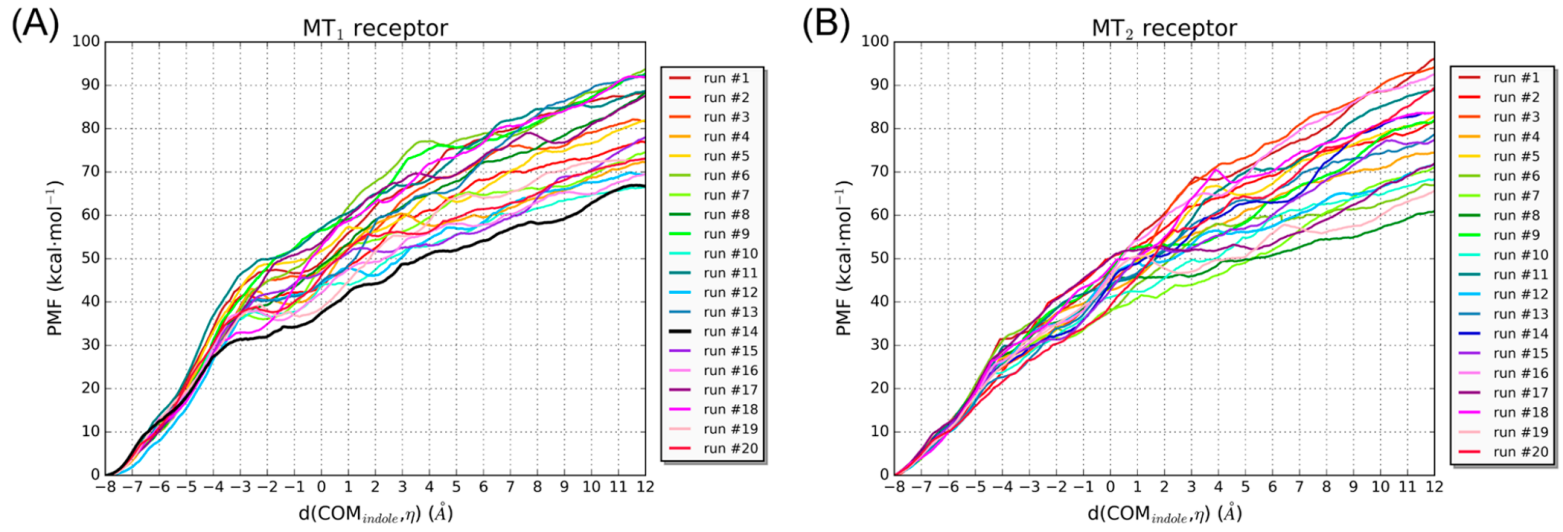
Work profiles from SMD simulations of 2-iodomelatonin unbinding from the MT_1_ (A) and MT_2_ (B) receptors through the lipophilic route. The ligand exits the binding site from the gap between TM helices IV and V. PMF profiles are reported over the distance *d*(*COM*_*indole*_, η) of the center of mass of the indole ring from a plane η comprising TM helices IV and V (see Figure 3 for the definition of plane η). A value of *d*(*COM*_*indole*_, η) = −8 Å corresponds to 2-iodomelatonin within the binding site in a crystal-like arrangement; for *d*(*COM*_*indole*_, η) = 12 Å, the ligand is immersed in the lipid bilayer, and for *d*(*COM*_*indole*_, η) from −3 to 3 Å, it is located between TM helices IV and V. The run with the lowest PMF in the MT_1_ receptor (run #14 in panel A, black line) was used to define reference configurations for PCV-US simulations.

To investigate the unbinding process of 2-iodomelatonin from the MT_1_ and MT_2_ receptors, nonequilibrium steered molecular dynamics (SMD) simulations^53^ were performed, in which the ligand was forced to leave the binding site and move to the membrane lipid bilayer through the gap between TM helices IV and V. To enable ligand unbinding, a harmonic restraint was applied to 2-iodomelatonin, which was forced to proceed toward the membrane following a specific direction, defined in the coordinate space as depicted in Figure 3. Twenty SMD simulations were performed for each receptor. 2-Iodomelatonin left the receptors following a different trajectory for each simulation, hampering the identification of defined routes and a direct comparison between MT_1_ and MT_2_ receptors. The work profiles obtained from the SMD simulations (Figure 5) have high energy content, not consistent with the experimental dissociation free energy and likely due to the attrition encountered by the ligand during the unbinding process. Even if it is not possible to estimate a reliable free-energy barrier from these simulations due to the nonequilibrium condition achieved through SMD,^65^ replicas of SMD provided preliminary information on the unbinding process. In all simulations, 2-iodomelatonin crossing through TM helices IV and V caused an increase in the distance between the same helices, which generally returned at the initial value as soon as the unbinding was completed. The major difference between MT_1_ and MT_2_ receptor simulations was related to the arrangement of Tyr187/200^5.38^ which spent more time in the open state during the unbinding of 2-iodomelatonin from the MT_1_ than from the MT_2_ receptor (Figure S8).

**Figure 6:**
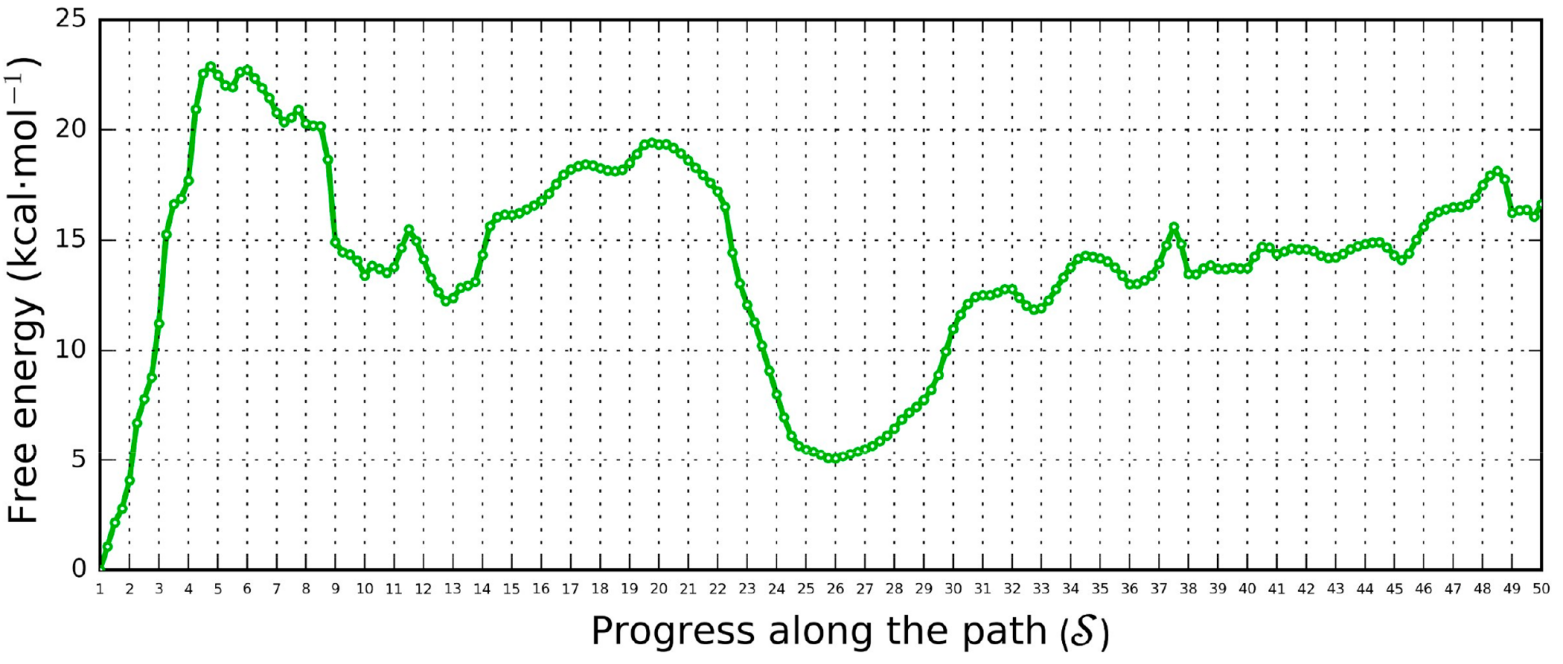
Free-energy profile of 2-iodomelatonin unbinding from the MT_1_ receptor from PCV-US simulations. The following ligand–receptor arrangements could be observed (see also Figure 7): i. the bound ligand ( = 1–5); ii. metastable arrangement of the ligand with the indole inserted between TM helices IV and V ( = 6–21); iii. the ligand interacting with the recognition site at the protein–membrane interface ( = 22–38); iv. the unbound ligand in the membrane bilayer ( = 39–50).

**Figure 7:**
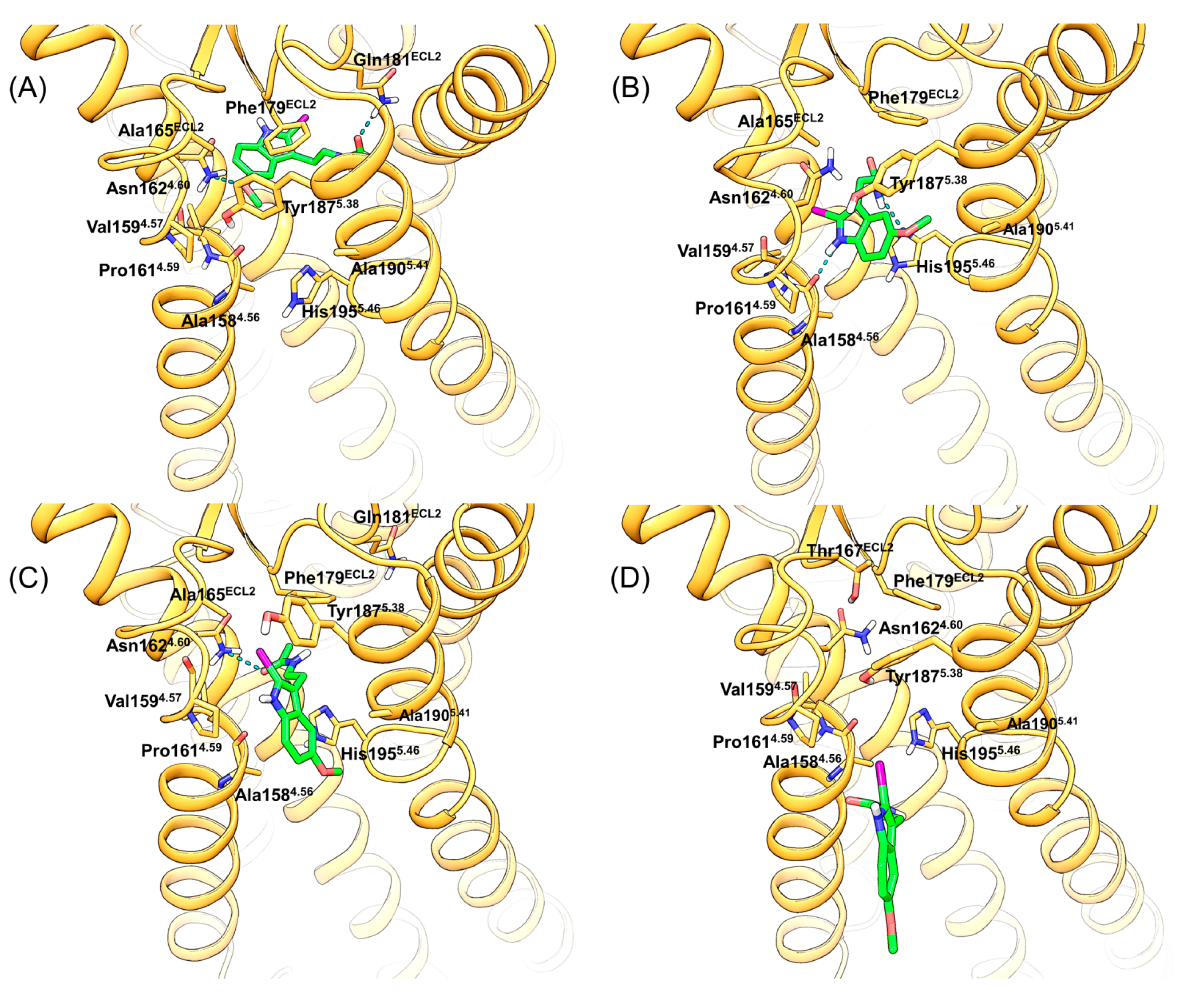
Representative ligand–receptor complexes during 2-iodomelatonin unbinding from the MT_1_ receptor in PCV-US simulations. The bound ligand at  = 1 (A); a metastable state captured during 2-iodomelatonin egress through the TM channel at = 13 (B); the ligand interacting with the putative recognition site at the protein–membrane interface corresponding to  = 26 (C); and the unbound ligand in the lipid bilayer at  = 50 (D).

Given the variability of free-energy estimation from SMD simulations, we decided to calculate the free-energy barrier for the 2-iodomelatonin receptor unbinding from a specific trajectory. Therefore, one of the unbinding trajectories obtained for the MT_1_-2-iodomelatonin complex was chosen (run #14, black line in Figure 5.A), having the lowest average work (∼28.1 kcal·mol^–1^) measured at a coordinate corresponding to the transition of the indole ring between the TM helices (*d*(*COM*_*indole*_, η) = 3 Å), which is expected to require high energy. This trajectory was employed for the definition of the path collective variable (PCV)^55^ through 50 reference configurations accounting for both ligand and receptor movements. The geometry of ligand heavy atoms and selected alpha carbons belonging to TM helices IV and V and to ECL2 (Figure 4) was included in the definition of the reference configurations. To estimate the unbinding free energy, PCV-US simulations were performed, with 50 ns of MD simulations restrained at the coordinates defining each  unitary value. A barrier of about 23 kcal·mol^–1^ ( = 4–6 in Figure 6) is encountered by the ligand to leave the bound state ( = 1, Figures 6 and 7.A). After that, the ligand arranges its indole ring between TM helices IV and V, with a transient decrease of free energy ( = 9–14). The indole nitrogen undertakes hydrogen bonds with Val159^4.57^ ( = 10) and Ala158^4.56^ ( = 13, Figure 7.B), favoring the rotation of the indole ring inside the TM channel. Another local minimum is found at  = 26 (Figure 7.C), where the ligand resides at the protein–membrane interface, in close proximity to the side chain of Tyr187^5.38^ in an open conformation. This minimum is separated by an association barrier of about 15 kcal·mol^–1^ from the intrahelix arrangements and is followed by a nearly plateau region corresponding to the unbound ligand in the membrane bilayer ( = 50, Figure 7.D).

The calculated barrier of about 23 kcal·mol^–1^ for 2-iodomelatonin dissociation from the MT_1_ receptor is close to the value of 25 kcal·mol^–1^ obtained from the experimental *k*_off_ of 2-[^125^I]-iodomelatonin at 25 °C.^25^ Although this value is an estimate for just one out of many possible unbinding trajectories, our simulation supports the feasibility of a lipophilic route for melatonergic ligands, demonstrating that there is at least one path going from the binding pose to the membrane core and passing through the TM helices IV and V, with an energy cost consistent with the experimental data. Moreover, this simulation revealed two aspects of the unbinding path toward the membrane that deserve attention, regarding the free-energy minimum at  = 26 and the role of Tyr187^5.38^.

**Figure 8:**
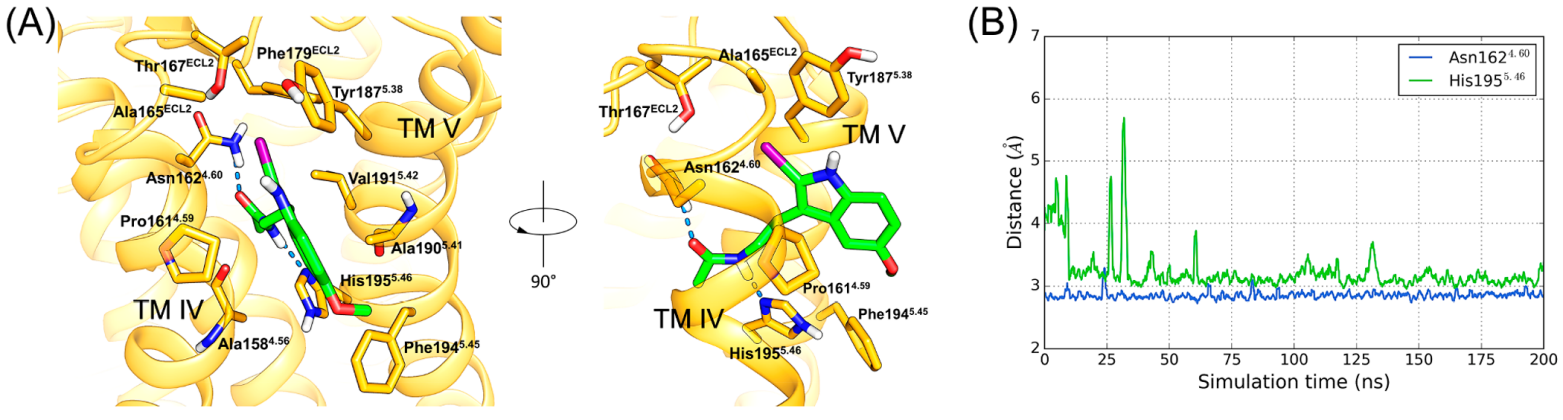
A recognition site for melatonergic ligands at the MT_1_ receptor interface with the membrane. A representative snapshot from a 200 ns-long molecular dynamics simulation of 2-iodomelatonin bound to the putative MT_1_ recognition site (A). Tyr187^5.38^ is in the open state, favoring ligand recruitment and crossing of TM helices IV and V. 2-Iodomelatonin undertakes stable hydrogen bonds (B) with Asn162^4.60^ and His195^5.46^ in the recognition site.

The energy minimum corresponding to  = 26 (Figure 7.C) can be envisaged as a recognition event favoring ligand recruitment from the membrane, prior to the binding process through the TM channel. In fact, an MD simulation of this MT_1_ receptor–ligand complex, with no restraint on the PCVs, showed stable interactions of the amide group with residues lining TM helices IV and V (Figure 8). The residues identified as counterparts for the amide group are Asn162^4.60^, which interacts with the methoxy oxygen of the ligand once it is accommodated within the binding site, and His195^5.46^ highly conserved in melatonin receptors, whose mutation in the MT_1_ subtype reduces protein expression^11^ and decreases melatonin binding affinity.^11,66^ During the simulation, the 2-iodo substituent of the ligand was accommodated close to the phenyl ring of the “gatekeeper” Tyr187^5.38^, which assumed an open-state conformation.

Spectroscopic measurements^67^ and metadynamics (MetaD) simulations^68^ had shown that, due to its physicochemical properties, melatonin is concentrated within the membrane in correspondence to the boundary between phospholipid polar heads and the lipophilic core formed by acyl chains. Thus, adsorption on the surface of TM helices IV and V could favor the access of a ligand to the orthosteric binding site through the channel between these helices. This can account for the potency gain observed for more lipophilic melatonin receptor ligands, and within this hypothesis, the design of novel ligands should consider physicochemical properties accounting for the partition of the compounds in the lipid bilayer.

### Tyr5.38 as a Molecular Determinant for Residence Time and Subtype Selectivity

**Figure 9:**
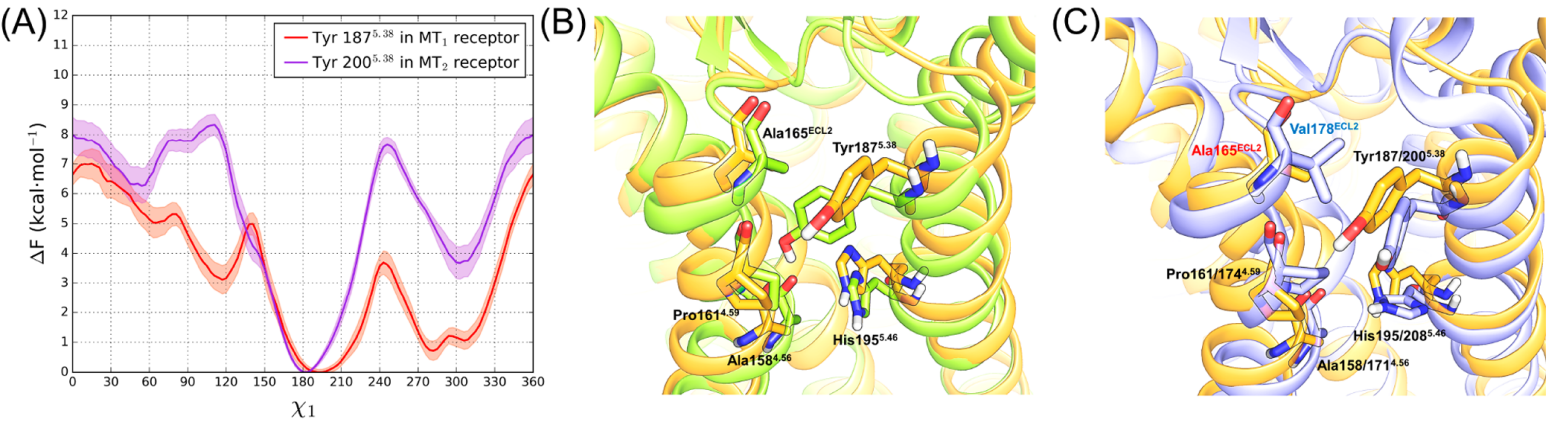
A greater propensity of Tyr187^5.38^ open state is observed in the MT_1_ receptor compared to the MT_2_ receptor. Free-energy profiles from 20 independent probability-averaged well-tempered MetaD simulations on a Tyr5.38 χ_1_ dihedral angle (A) evidence a difference of about 1 kcal·mol^–1^ between the open (χ_1_ ≈ 300°) and closed (χ_1_ ≈ 180°) states in the MT_1_ receptor (red line) and of about 4 kcal·mol^–1^ in the MT_2_ receptor (purple line). The shaded region of the two curves represents the SEM of the free energy. Superposition of representative snapshots extracted from monodimensional MetaD simulations of (B) the open and closed states of Tyr187^5.38^ in the MT_1_ receptor (open conformation: orange; closed conformation: green) and (C) the open state of Tyr5.38 in the MT_1_ (orange) and MT_2_ receptors (white). Ala165^ECL2^ (red label) is present in the MT_1_ receptor, and Val178^ECL2^ (blue label) is present in the MT_2_ receptor.

Tyr187/200^5.38^, already observed in an open and a closed conformation in the crystal structures of MT_1_ and MT_2_ receptors, respectively, emerges as a critical residue influencing the behavior of melatonin receptors in our simulations. While during plain MD simulations of MT_1_ and MT_2_ receptor-agonist complexes it assumed a closed conformation for both receptors, in SMD simulations, Tyr187/200^5.38^ preferred an open conformation during ligand unbinding from the MT_1_ receptor (Figure S8). Additionally, in the PCV-US simulations, in which the reference geometries were retrieved from an SMD trajectory with Tyr187^5.38^ in the open state during ligand unbinding, the side chain is open, while the ligand exits from the MT_1_ receptor binding site and returns to the closed state once the ligand is fully unbound (Figure S9). To quantify the conformational preference of Tyr5.38 in the two receptors, we performed well-tempered MetaD simulations^63^ using a Tyr5.38 χ_1_ dihedral angle as the collective variable (*CV*). Since MetaD simulations provided highly variable results and did not converge at reasonable simulation times, for each receptor subtype, we performed 20 replicas that were combined in the average free-energy profiles shown in Figure 9.A. For the MT_1_ receptor, the open (χ_1_ ≈ 300°) and closed (χ_1_ ≈ 180°) states (Figure 9.B) have similar free-energy values, with the closed state only slightly preferred for ∼1 kcal·mol^–1^; in the MT_2_ receptor, the closed state was predominant for around 4 kcal·mol^–1^. These results were confirmed by 1 μs-long bidimensional well-tempered MetaD simulations, with Tyr5.38 χ_1_ and χ_2_ dihedral angles as *CV*s (Figures S6 and S7), showing similar energies for the open and closed states in the MT_1_ receptor and a marked preference for the closed state in the MT_2_ receptor.

A comparison of MT_1_ and MT_2_ structures with Tyr5.38 in the open state (Figure 9.C) shows that the aromatic ring faces different amino acids from ECL2, namely Ala165 in MT_1_ and Val178 in MT_2_ receptors. The bulkier MT_2_ Val178 hampers the open conformation of Tyr5.38 and is likely one of the causes for the prevalence of the closed conformation. Results from MD simulations are consistent with mutagenesis data since mutation of Tyr5.38 to alanine led to a greater decrease of [^3^H]-melatonin residence time at the MT_2_ than at the MT_1_ receptor (30- vs 4-fold decrease).^12^

Very recently, the three-dimensional structure of the MT_1_ receptor in complex with the agonist ramelteon and G_i_ protein has been reported.^13^ Superposition of the active receptor conformation with the previous inactive, agonist-bound ones highlights a high degree of conservation of the extracellular side of the receptor and of agonist arrangement, as it has already been observed for ligands of other class A GPCRs.^69^ Wide conformational changes affect the intracellular side of TM helices VI and VII as a consequence of the interaction with the G_i_ protein, while the backbone of TM helices IV and V overlaps the coordinates of the previous X-ray structures. The side chain of Tyr187^5.38^ assumed a closed conformation, analogous to the one observed in the MT_2_ crystal structures and corresponding to the global minimum of the MetaD simulations performed on the MT_1_ receptor (Figures 9.A and 9.B). This experimental finding corroborates the possibility for Tyr187^5.38^ to access two alternative states, further providing evidence for its role as a “gatekeeper” residue at the MT_1_ receptor.

**Figure 10:**
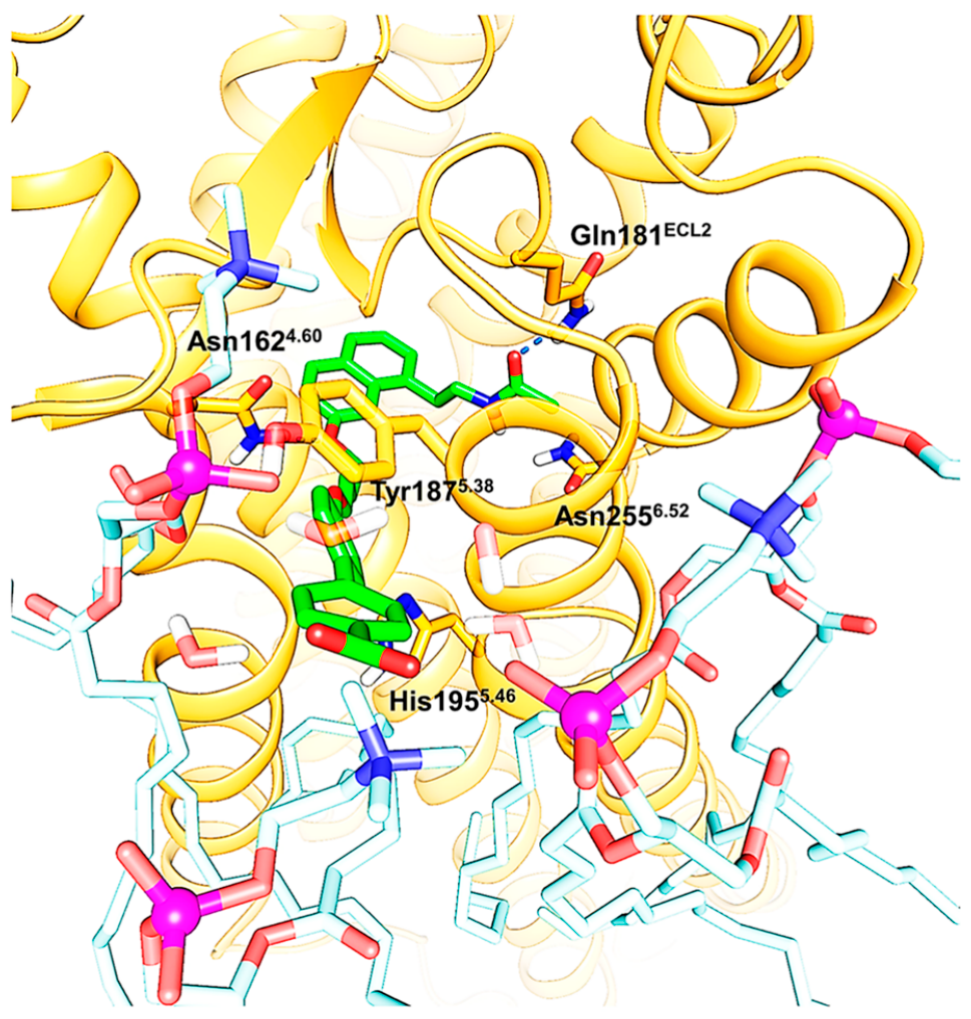
MT_1_-selective compound **1** interacts with membrane cholines. A representative snapshot from the molecular dynamics simulation of the agomelatine derivative **1** in complex with the MT_1_ receptor is shown. The open conformation of the Tyr187^5.38^ side chain widens the lateral channel and favors the orientation of the biphenyl-carboxylate toward the membrane-solvent interface, allowing interactions with choline polar heads.

While many compounds had shown good to excellent selectivity for the MT_2_-receptor subtype, the molecular determinants for high MT_1_ selectivity still need to be clearly defined.^8^ The propensity of Tyr5.38 to assume the open state during ligand unbinding from the MT_1_ receptor allows the generation of transient configurations in which the ligand interacts with residues at the interface between TM helices IV and V. While such interactions were observed during the unbinding simulation of 2-iodomelatonin from the MT_1_ receptor, it can be speculated that the side chain of Tyr5.38 in its open conformation could also interact with MT_1_-selective ligands, favoring their binding to the receptor. The long and lipophilic substituents replacing the methoxy group of melatonin in MT_1_-selective ligands could play such a role. In fact, docking studies of an MT_1_-selective agomelatine dimer had suggested the possibility to occupy both the orthosteric binding site and the region between TM helices IV and V, protruding through the channel toward the membrane lipids.^11^ Moreover, a biphenyl agomelatine derivative with a terminal carboxylic group (Figure 1, compound **1**, *K*_*i*_(MT_1_) = 0.55 nM and *K*_*i*_(MT_2_) = 51.30 nM) was reported to be more selective for the MT_1_ receptor than its neutral analogues.^10^ A 200 ns-long molecular dynamics simulation of the complex between compound **1** and the MT_1_ receptor showed that, starting from a docking pose with the biphenyl substituent protruding toward the inner portion of the lipid bilayer (Figure S1), the biphenyl was readily accommodated close to the open Tyr187^5.38^, with the terminal carboxylate group interacting with the choline trimethylammonium heads either directly or through bridging water molecules placed at the membrane-solvent interface (Figure 10). This simulation supports the hypothesis that interaction of ligand portions with the open Tyr187^5.38^ can favor MT_1_ selectivity.

## Conclusions and Future Perspective

We investigated the feasibility of a lipophilic route for melatonin receptor ligands that can transit from the orthosteric binding site, located in the 7-TM bundle, to the membrane through a lateral channel between TM helices IV and V. Simulation of the unbinding process for 2-iodomelatonin in complex with the MT_1_ receptor furnished a calculated energy barrier of about 23 kcal·mol^–1^, consistent with experimental *k*_off_ of the radiolabeled ligand,^25^ thus sustaining the lipophilic route as a feasible route for ligand entrance and egress from the binding site. The unbinding simulation captured 2-iodomelatonin in contact with a recognition site at the entrance of the receptor, generated by amino acids from TM helices IV and V. Similarly to what had been proposed in computational works on other ligands,^70^ the interaction of 2-iodomelatonin with this region might assist and promote ligand recruitment and dissociation, forming transient but energetically favored arrangements. These additional binding sites have been proposed as targets for allosteric receptor modulators, as in the case of the β_2_-adrenergic receptor.^71^ Our simulation also supports the hypothesis that in the MT_1_ receptor this region could serve as a binding site for bitopic ligands which, besides interacting with the orthosteric site, are able to engage an allosteric region. Occupation of the allosteric site, as proposed for agomelatine derivatives **1**, would provide selectivity for the MT_1_ receptor and, potentially, increased binding affinity,^23,72^ with ligands able to interact not only with TM helices but also with components of the membrane. Knowledge of the MT_1_ structure and dynamics will likely help the design of subtype selective agonists and antagonists which are currently limited in both degree of selectivity and structural diversity.^8,9^ The external recognition region could also be exploited by dual-acting compounds, in which the pharmacophore for the second target can either interact with the allosteric site or remain at the lipid interface.^73^

The major difference observed in molecular dynamics simulations of the two receptor subtypes was the behavior of Tyr5.38. This residue showed a significant preference for a closed state in the MT_2_ receptor, in which its side chain interacts with TM helix IV, while in the MT_1_ receptor, the closed and open states were almost equally populated. In the open state, Tyr5.38 points toward the membrane and enables ligand interaction with the cited recognition site, promoting ligand access and egress from the receptor.

These results were obtained with simulations performed on systems built as described in the Methods section, and different conditions, such as protonation states, might lead to different results. In fact, it has already been observed that alternative protonation states alter the behavior of proteins during simulations.^74^ In this work, the protonation state of histidine residues was chosen on the basis of the polar surroundings and maintained during the simulation, but it cannot be excluded that different protonation or tautomeric states can significantly affect the energetics of simulated systems.

Another limitation of the present work is the lack of simulations performed with receptors mutants. This is mainly due to the high variability of the unbinding trajectories in steered MD runs, which limits the precision of quantitative comparisons. On the other hand, the mutation of residues lining the lipophilic route had given results that are qualitatively consistent with our computational study. Thus, mutation of Tyr5.38 to alanine reduces *k*_off_ of [^3^H]-melatonin for the MT_1_ receptor, while mutations of Ala4.56 or Ala5.41, which are close to the unbinding trajectories (Figures 7 and 8), to bulkier residues increase [^3^H]-melatonin *k*_off_^12^ or reduce agonist functional activity.^11^

Different aspects of melatonin receptors functions and activity still need to be investigated, such as the relationship between ligand structure, tissue distribution, and biased signaling,^75^ the impact of receptor dimerization/oligomerization on activity,^76^ or the determinants for receptor subtype selectivity. The application of atomistic simulations coupled to enhanced sampling techniques will be a valuable tool to unveil aspects and behaviors of melatonin receptors that cannot be straightforwardly derived from observation of crystal structures of receptor–ligand complexes. Transient binding sites, metastable geometries, and associated probabilities identified through molecular simulations could not only provide a hypothesis for observed biological events but also drive the design of new ligands with customized pharmacological properties.

**Data and Software Availability**. All data and software are available upon reasonable request to the corresponding author. GROMACS (https://www.gromacs.org/) is an open-source and free MD package. PLUMED (https://www.plumed.org/) is an open-source plugin for MD.

## Supporting Information Available

Detailed information about systems preparation and design of unbinding *CV*; pulling simulations for PCV optimization; analysis of PCV-US simulations; and supplementary results for Tyr187/200^5.38^ conformational analysis (PDF)
GROMACS and PLUMED input files to run SMD, PCV-US and monodimensional and bidimensional MetaD simulations (ZIP)
