# Supplementary material for: Free-Energy Simulations Support a Lipophilic Binding Route for Melatonin Receptors
Source: J Chem Inf Model. 2021 Dec 21;62(1):210–22. doi: 10.1021/acs.jcim.1c01183 (PMC8757440; doi:10.1021/acs.jcim.1c01183)
Supplement: Supplementary file 1 — ci1c01183_si_001.pdf [file ci1c01183_si_001.pdf]

Supporting Information for:

Free-energy simulations support a lipophilic binding route for melatonin receptors

Gian Marco Elisi[1], Laura Scalvini[1], Alessio Lodola[1], Marco Mor[1,2], Silvia Rivara[1]*

[1] Dipartimento di Scienze degli Alimenti e del Farmaco, Università degli Studi di Parma, Parco Area delle Scienze 27/A, I-43124 Parma, Italy

[2] Microbiome Research Hub, University of Parma, I-43124 Parma, Italy

Corresponding Author

* Dipartimento di Scienze degli Alimenti e del Farmaco, Università degli Studi di Parma, Parco Area delle Scienze 27/A, I-43124 Parma, Italy. Phone: +39 0521 905059. E-mail: marco.mor@unipr.it

S1. Docking of compound 1 in the MT_1 receptor

The crystal structure of the MT_1 receptor in complex with 2-phenylmelatonin (PDB id 6ME3)[1] was used for docking calculations after deletion of the co-crystallized ligand. The docking grid was centered on 2-phenylmelatonin, defining a bounding box of 20x10x10 Å and an enclosing box of 40x30x30 Å. Ligand docking was performed with Glide 7.9[2,3] in standard precision mode and with default scaling factors,[2] setting MAXKEEP and MAXREF parameters, which control the number of poses to retain after the rough scoring stage and the number of poses to refine, to 50,000 and 4000, respectively. The scoring window cutoff was increased to 1000 to widen the selection of the initial poses for the rough scoring stage. The best ligand pose according to the GScore scoring function was merged into the protein structure and the complex (Figure S1) was energy-minimized to a gradient of 0.01 $kJ \cdot mol^{-1} \cdot Å^{-1}$ with OPLS3e force field[4] implemented in MacroModel 12.0[5] in an implicit water solvation model,[6] using the Polak-Ribière conjugate gradient method.[7]

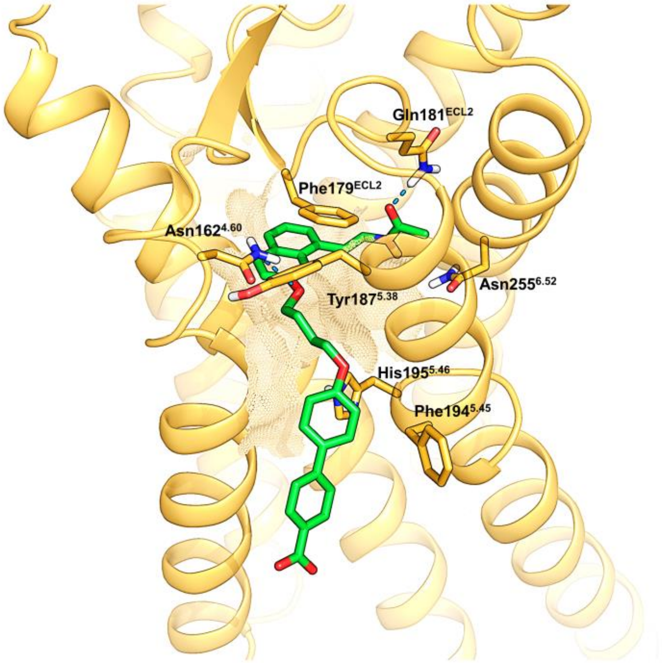

Figure S1. Docking pose of compound **1** in the MT_1 receptor. The naphthalene nucleus resides in the orthosteric binding site, the amide side chain and the methoxy oxygen interact with Gln181^{ECL2} and

Asn162$^{4.60}$, respectively, as observed for co-crystallized ligands.[1] The biphenyl-carboxylate moiety is accommodated at the protein-membrane interface and protrudes through the channel between TM helices IV and V via the oxybutyloxy spacer.

S2. Minimization and equilibration protocol

The systems were minimized using the sander module implemented in Amber16[8] with three minimization cycles of 5,000 steps, each one passing from the steepest descent method to the conjugate gradient, and with protein backbone restrained with a constant of 50 $kcal \cdot mol^{-1} \cdot Å^{-2}$. The coordinates and the topology files of the minimized structures were then converted in the Gromacs coordinates and topology input files with the Acpype parser.[9]

Systems equilibration of the minimized complexes comprised different stages:

- 4.0 ns in NVT ensemble,[10] heating from 10 to 200 K with a timestep of 1 fs, with restrained protein and ligand heavy atoms. The membrane was relaxed in the last 2.0 ns, with only heteroatoms of phosphocholine restrained;
- 4.0 ns in NPT ensemble,[10] heating from 200 to 298 K with a timestep of 1 fs, with restrained protein and ligand heavy atoms. During the first ns, phosphocholine heteroatoms were also restrained;
- 12.0 ns in NPT ensemble, with protein and ligand heavy atoms progressively relaxed, until only the alpha carbons were restrained with a spring constant of 0.1 $kcal \cdot mol^{-1} \cdot Å^{-2}$.[11,12,13,14]

These steps were followed by two NVT[13,14] stages restraining the positions of alpha carbons to those of the crystal structures:

- 100.0 ns in NVT ensemble, with alpha carbons restrained with a spring constant of 0.1 $kcal \cdot mol^{-1} \cdot Å^{-2}$. The output structure of this NVT stage was used as input structure for bidimensional metadynamics (Paragraph S7).

- 100.0 ns in NVT ensemble, with only 89 selected backbone carbons (see Paragraph S3 for backbone atoms selection) restrained with a spring constant of 0.1 kcal·mol^{-1}·Å^{-2}. The output structure of this NVT stage was used as input structure for all the remaining enhanced sampling simulations and for MD simulation of the MT_1 receptor in complex with compound **1**.

S3. Definition of restrained backbone carbons

89 carbons were restrained in MT_1 and MT_2 receptors, comprising 5 carbons on the truncated N-terminal tip (the carbonyl carbon of the acyl-cap and alpha carbons of the first four residues) and 84 alpha carbons belonging to three consecutive turns in the intracytoplasmic side of each TM helix (12 alpha carbons for each helix). The following alpha carbons were restrained:

MT_1 receptor		MT_2 receptor	
N-term:	Pro23-Leu26	N-term:	Pro36-Val39
TM I:	Leu43-Arg54	TM I:	Val56-Arg67
TM II:	Ile64-Val75	TM II:	Leu77-Val88
TM III:	Ile123-Lys134	TM III:	Ile136-Ala147
TM IV:	Ser144-Thr155	TM IV:	Thr157-Thr168
TM V:	Tyr207-Arg218	TM V:	Tyr220-Arg231
TM VI:	Arg235-Leu246	TM VI:	Arg248-Ile259
TM VII:	Asn287-Leu298	TM VII:	Asn300-Leu311

S4. Unbinding CV for steered molecular dynamics: definition of plane η

Plane η is defined through the coordinates of three centers of mass, H_1, H_2 and H_3, defined in counterclockwise order, observing from the outside of the receptor, as in Figure 2 of the main text. The centers of mass are related to the following alpha carbons:

MT_1 receptor		MT_2 receptor	
H1:	Ser144-Thr155	H1:	Thr157-Thr168
H2:	Tyr207-Arg218	H2:	Tyr220-Arg231
H3:	$\overbrace{\text{Leu156} - \text{Leu163}}^{TM\ IV}, \overbrace{\text{Thr188} - \text{His195}}^{TM\ V}$	H3:	$\overbrace{\text{Val169} - \text{Phe176}}^{TM\ IV}, \overbrace{\text{Thr201} - \text{His208}}^{TM\ V}$

S5. Path CVs (PCVs) optimization: technical details

Path optimization[15] was performed through four consecutive steered molecular dynamics (SMD) simulations[16] of 30.0 ns in which a harmonic restraint of 50 kcal·mol^{-1} was applied on the $\mathcal{S}$ CV, restricting the access to configurations of the system distant from the reference frameset by restraining the exploration of the $\mathcal{Z}$ CV with a force constant of 1,000 kcal·mol^{-1}·Å^{-4} at $\mathcal{Z}$=1 Å^2. In these simulations, λ was tuned to assure a smooth transition between coordinates of the system corresponding to reference configurations with the highest inter-frame distance in the region of the CV ($\mathcal{S}$=1-38), in which the ligand interacts with the receptor (λ_1=1.05Å^{-2}, λ_2=1.87Å^{-2}, λ_3=2.00Å^{-2}, λ_4=2.08Å^{-2}).

Between each pair of the four SMD-PCV runs, the reference configurations were iteratively updated using in-house scripts. A procedure using the Catmull-Rom splines method[17] was set up to generate additional configurations to obtain a smoother guess path, by interpolating the SMD trajectory through the insertion of ten intermediate configurations between each pair of consecutive SMD frames. The 50 reference configurations were then extracted, on the basis of an RMSD-distance matrix calculated on the whole frameset, through an operation based on the Nudged Elastic Band (NEB) approach;[18] in this procedure, the Monte Carlo sampling was used to search for the best sequence of configurations describing transition states regions. 10 iterations of the NEB algorithm were performed using a Metropolis coefficient of 10,000, keeping the endpoints (first and last reference configurations) fixed. After every SMD-PCV simulation, the set of frames thus obtained was employed as the new (and refined) reference path for a subsequent SMD-PCV run. In three consecutive runs, the work profile stabilized around similar values (Figure S2). The reference configurations within $\mathcal{S}$ ranges 2-5 and 38-50 were further optimized through targeted dynamics simulations setting as a collective variable the difference of the RMSD between the first and last configuration of each range.[19]

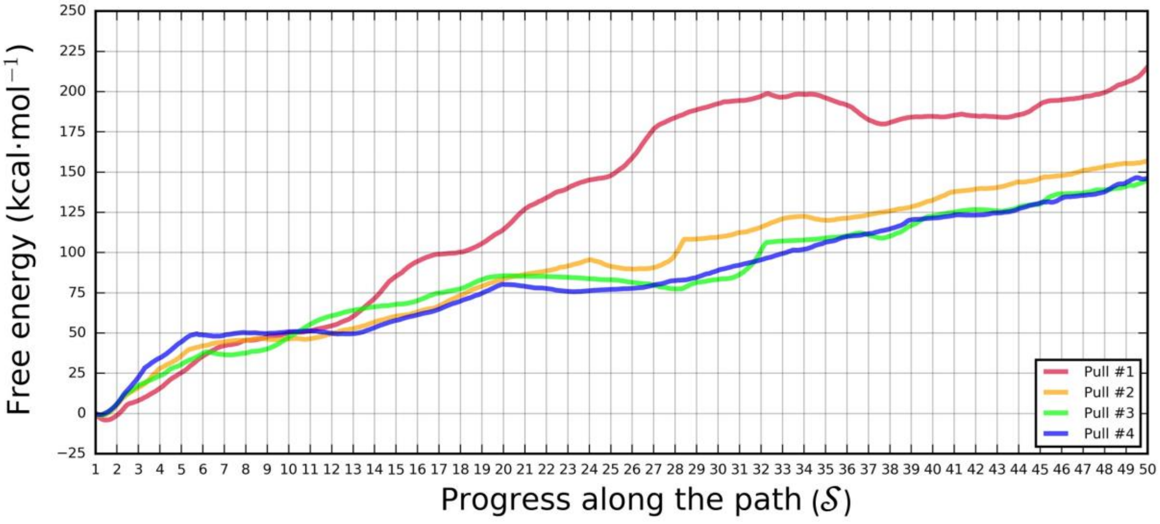

Figure S2. Work profiles of the four consecutive SMD-PCV simulations performed to optimize the path reference configurations.

S6. Analysis of PCV-US simulations

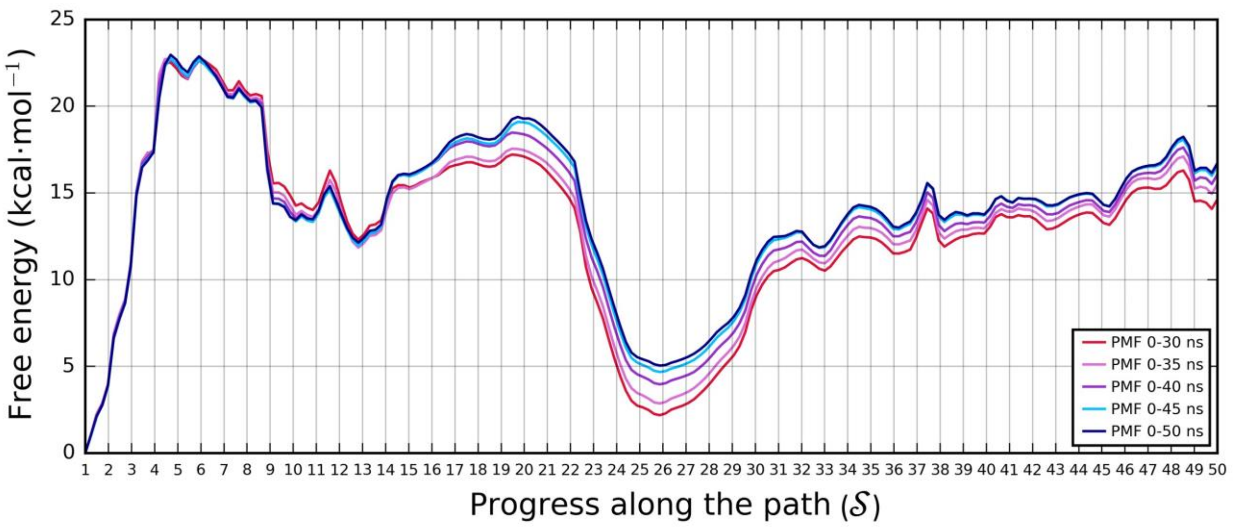

Figure S3. Free-energy profile obtained for PCV-US simulations as a function of the simulation time of each US window. Potential of mean force (PMF) curves were calculated using the WHAM algorithm,[20,21] by renormalizing the probabilities for each window into a single composite probability.

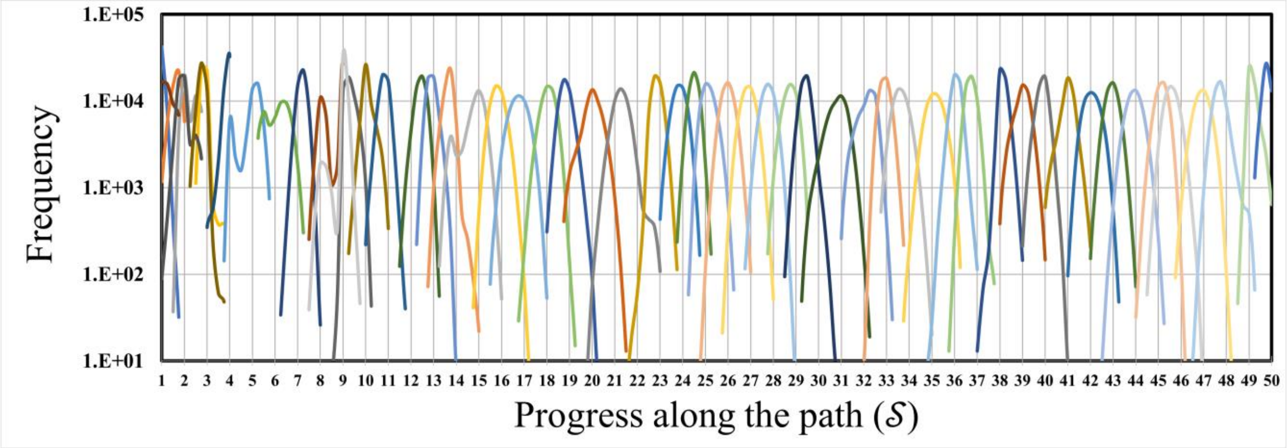

Figure S4. Distribution (log scale) of $\mathcal{S}$ values of PCV-US simulations. The value of the $\mathcal{S}$ coordinate was registered every ps for each simulation.

Qualitative agreement of the US windows *a priori* probabilities

The global PMF, given by the contributions of all the US windows employed in the WHAM calculation, is a free-energy profile over the coordinate $\mathcal{S}$, divided in intervals j:

$$PMF_j = -k_B T \cdot \ln p_{0_j} + \text{F} \qquad \text{(S1)}$$

where the constant F is fixed to have PMF($\mathcal{S}$=1) = 0 kcal·mol^{-1}.

The reliability of PMF was evaluated through the qualitative agreement between *a priori* probabilities of neighboring US windows (Figure S5). The *a priori* probability p_{0_j} is related to the biased probability p_{ij} that the ith simulation falls into the jth interval according to the following relationship:

$$P_{ij} = P_{0_j} \cdot c_{ij} \cdot f_i \qquad \text{(S2)}$$

where c_{ij} represents the probability distortion due to the bias potential and f_i is a normalization coefficient.

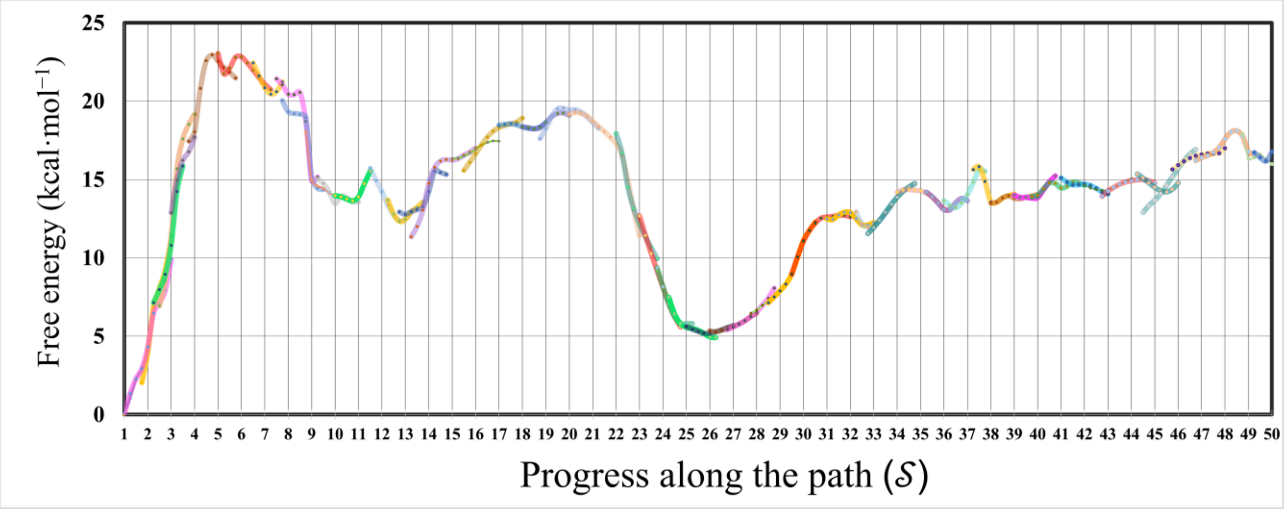

Figure S5. Dissection of the global potential of mean force (PMF) into the free-energy profiles of the PCV-US windows, scaled as free-energy differences with respect to $\mathcal{S}$=1 (bound state).

S7. Well-tempered metadynamics simulations on Tyr187/200$^{5.38}$

Table S1. Parameters adopted for monodimensional and bidimensional well-tempered metadynamics simulations on Tyr187/200$^{5.38}$ dihedral angles.

Parameters	1D	2D
$\boldsymbol{\sigma\ (\chi_1)}$	3 °	5 °
$\boldsymbol{\sigma\ (\chi_2)}$	--	10 °
$\boldsymbol{h_0}$	0.2 kcal·mol^{-1}	0.2 kcal·mol^{-1}
$\boldsymbol{\tau}$	3 ps	5 ps
$\boldsymbol{\gamma}$	15	30
**restraints**	89 atoms (see Paragraph S3)	all alpha carbons

Bidimensional well-tempered metadynamics simulations

Bidimensional well-tempered metadynamics simulations[22] were performed with restrained receptor alpha carbons. χ_2 dihedral angle was compactified in a [0,π] interval with a Heaviside function to avoid the exploration of symmetric degrees of freedom. FES are shown after 1.0 and 1.1 μs of simulation (Figure S6). For each system, the exploration of different minima across the CVs and the time-evolution of potentials deposition was assessed (Figure S7).

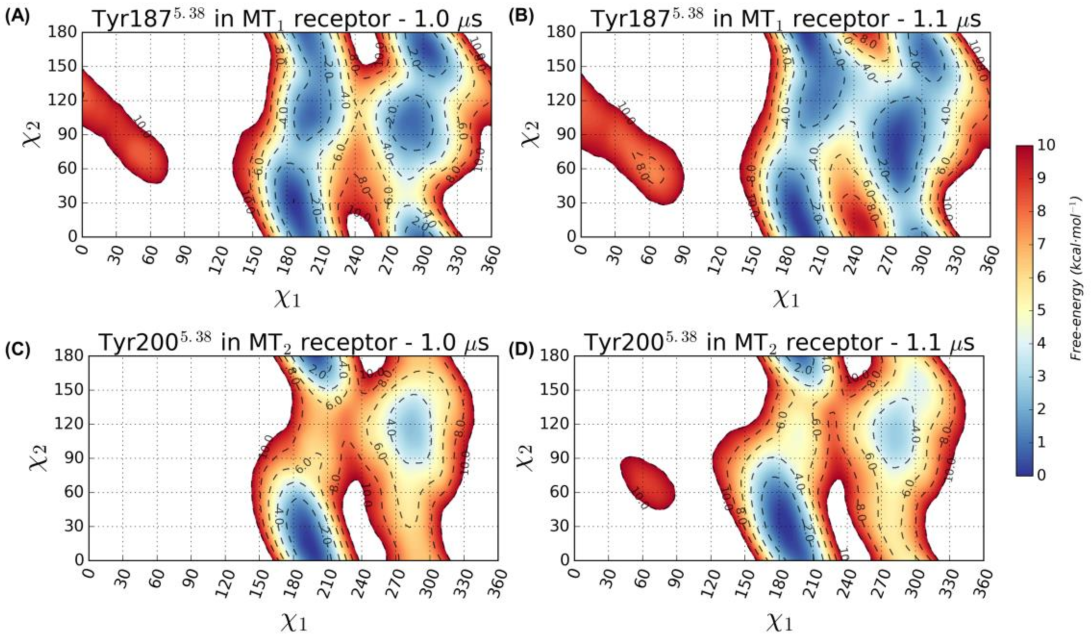

Figure S6. Free-energy surfaces for Tyr5.38 side chain after 1.0 (**A** and **C**) and 1.1 µs (**B** and **D**) of well-tempered metadynamics simulations performed on dihedral angles χ_1 and χ_2 of the MT_1 (**A** and **B**) and MT_2 (**C** and **D**) receptors in complex with 2-iodomelatonin.

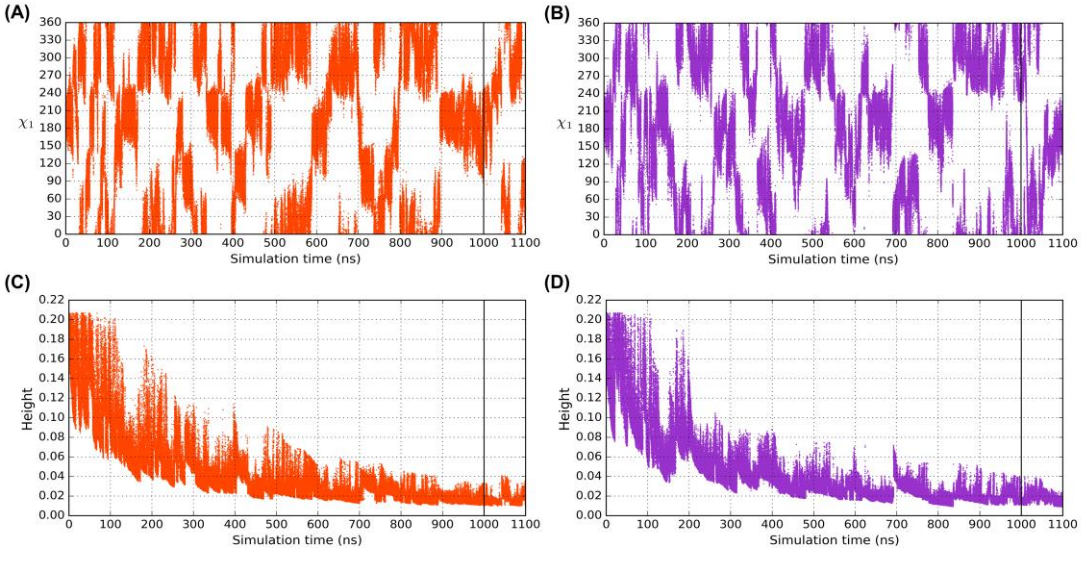

Figure S7. Time-evolution of the values assumed by the χ_1 dihedral angle in MT_1 (**A**) and MT_2 (**B**) receptors in complex with 2-iodomelatonin. χ_2 dihedral angle is not shown as it is a

completely diffusive CV. Time-evolution of the height of the Gaussian potentials deposited in the two systems (**C** and **D**).

S8. Analysis of Tyr187/200$^{5.38}$ side chain conformation during ligand unbinding

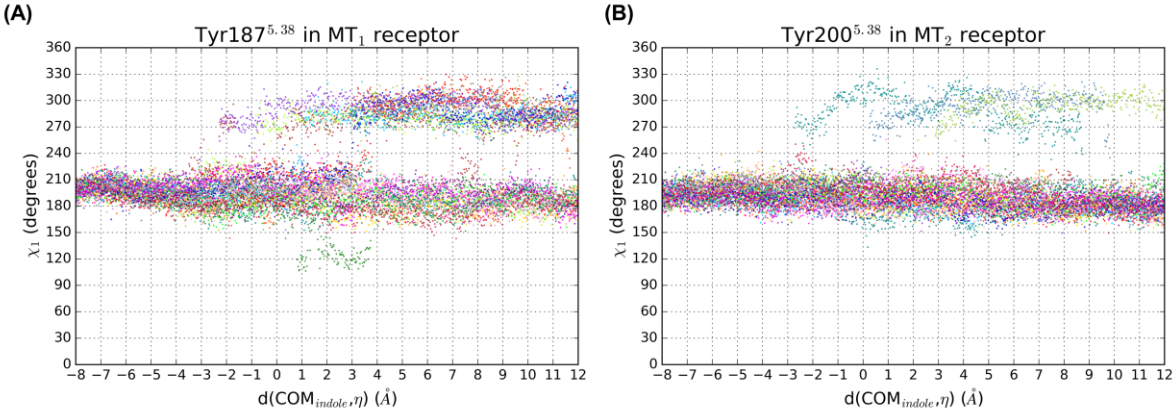

Figure S8. Conformation of Tyr187/200$^{5.38}$ in SMD simulations. Values of Tyr5.38 χ_1 dihedral angle monitored in the twenty SMD simulations during the unbinding of 2-iodomelatonin from the MT$_1$ (**A**) and the MT$_2$ (**B**) receptors. Each color represents a different simulation. The open state of Tyr5.38 side chain (270°<χ_1<300°) is more populated in simulations of ligand exiting from the MT$_1$ receptor than from the MT$_2$ receptor. χ_1 dihedral angle assumed values around 300° in ten out of twenty simulations of the MT$_1$-receptor complex, and in 3 simulations of the MT$_2$-receptor complex.

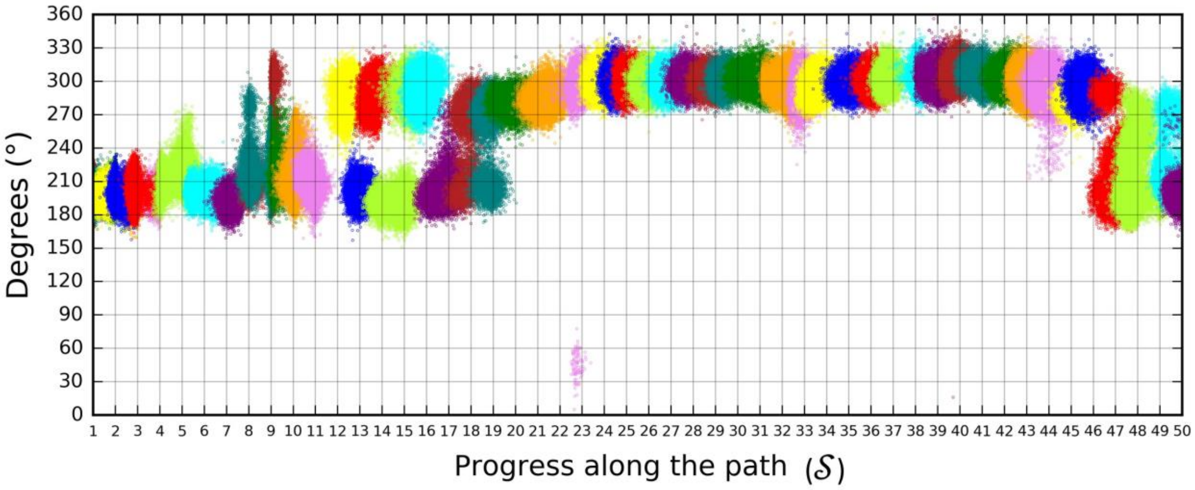

Figure S9. Conformation of MT_1 Tyr187$^{5.38}$ in PCV-US simulations. Values of Tyr187$^{5.38}$ χ_1 dihedral angle are monitored every 2 ps in PCV-US simulations of 2-iodomelatonin unbinding from the MT_1 receptor. Each color represents an independent simulation of 50 ns.

References

1 Stauch, B.; Johansson, L. C.; McCorvy, J. D.; Patel, N.; Han, G. W.; Huang, X.; Gati, C.; Batyuk, A.; Slocum, S. T.; Ishchenko, A.; Brehm, W.; White, T. A.; Michaelian, N.; Madsen, C.; Zhu, L.; Grant, T. D.; Grandner, J. M.; Shiriaeva, A.; Olsen, R. H. J.; Tribo, A. R.; Yous, S.; Stevens, R. C.; Weierstall, U.; Katritch, V.; Roth, B. L.; Liu, W.; Cherezov, V. Structural basis of ligand recognition at the human MT_1 melatonin receptor. *Nature* **2019**, 569, 284-288.

2 Friesner, R. A.; Banks, J. L.; Murphy, R. B. Glide: A new approach for rapid, accurate docking and scoring. 1. Method and assessment of docking accuracy. *J. Med. Chem.* **2004**, 47, 1739–1749.

3 *Schrödinger Release 2018-2: Glide 7.9*, Schrödinger, LLC, New York, NY, 2018.

4 Roos, K.; Wu, C.; Damm, W.; Reboul, M.; Stevenson, J. M.; Lu, C.; Dahlgren, M. K.; Mondal, S.; Chen, W.; Wang, L.; Abel, R.; Friesner, R. A.; Harder, E. D. OPLS3e: Extending force field coverage for drug-like small molecules. *J. Chem. Theory Comput.* **2019**, 15, 1863–1874.

5 *Schrödinger Release 2018-2: MacroModel 12.0*, Schrödinger, LLC, New York, NY, 2018.

6 Still, W. C.; Tempczyk, A.; Hawley, R. C. Semianalytical treatment of solvation for molecular mechanics and dynamics. *J. Am. Chem. Soc.* **1990**, 112, 6127–6129.

7 Polak, E.; Ribière, G. Note sur la convergence de méthodes de directions conjuguées. *Revue Française Inf. Rech. Opér. Sér. rouge* **1969**, 16, 35–43.

8 Case, D. A.; Betz, R. M.; Cerutti, D. S.; Cheatham, III, T. E.; Darden, T. A.; Duke, R. E.; Giese, T. J.; Gohlke, H.; Goetz, A. W.; Homeyer, N.; Izadi, S.; Janowski, P.; Kaus, J.; Kovalenko, A.; Lee, T. S.; LeGrand, S.; Li, P.; Lin, C.; Luchko, T.; Luo, R.; Madej, B.; Mermelstein, D.; Merz, K. M.; Monard, G.; Nguyen, H.; Nguyen, H. T.; Omelyan, I.; Onufriev, A.; Roe, D. R.; Roitberg, A.; Sagui, C.; Simmerling, C. L.; Botello-Smith, W. M.; Swails, J.; Walker, R. C.; Wang, J.; Wolf, R. M.; Wu, X.; Xiao, L.; Kollman P. A. AMBER 2016, University of California, San Francisco 2016.

9 Sousa da Silva, A. W.; Vranken, W. F. ACPYPE - AnteChamber PYthon Parser interfacE. *BMC Res. Notes* **2012**, 5, 367.

10 Berendsen, H. J. C.; Postma, J. P. M.; van Gunsteren W. F.; Di Nola, A.; Haak, J. R. Molecular dynamics with coupling to an external bath. *J. Chem. Phys.* **1984**, 81, 3684-3690.

11 Nosé S. A Molecular Dynamics Method for Simulations in the Canonical Ensemble. *Mol. Phys.* **1984**, 52, 255-268.

12 Hoover WG. Canonical dynamics: Equilibrium phase-space distributions. *Phys. Rev. A* **1985**, 31, 1695.

13 Parrinello, M.; Rahman, A. Polymorphic transitions in single crystals: a new molecular dynamics method. *J. Appl. Phys.* **1981**, 52, 7182-7190.

14 Nose, S.; Klein, M. L. Constant pressure molecular dynamics‘ for molecular systems. *Mol. Phys.* **1983**, 50, 1055-1076.

15 Branduardi, D.; Gervasio, F. L.; Parrinello, M. From A to B in free energy space. *J. Chem. Phys*. **2007**, 126, 054103.

16 Grubmüller, H.; Heymann, B.; Tavan, P. Ligand Binding: Molecular Mechanics Calculation of the Streptavidin-Biotin Rupture Force. *Science* **1996**, 271, 997-999.

17 Catmull, E.; Rom, R. A class of local interpolating splines. In Computer aided geometric design, 1st ed..; New York: Academic Press. In: Barnhill R. E. & Riesenfeld R. F., 1974; pp. 317–332.

18 Jónsson, H.; Mills, G.; Jacobsen, K. W. Classical Dynamics in Condensed Phase Simulations. In: B. J. Berne, G. Ciccotti, and D. F. Coker; World Scientific, Singapore, 1998; p. 385-404.

19 Favia, A. D.; Masetti, M.; Recanatini, M.; Cavalli, A. Substrate binding process and mechanistic functioning of type 1 11β-hydroxysteroid dehydrogenase from enhanced sampling methods. *PLoS One* **2011**, 6, e25375.

20 Kumar, S.; Rosenberg, J. M.; Bouzida, D.; Swendsen, R. H., Kollman, P. A. The weighted histogram analysis method for free-energy calculations on biomolecules. I. The method. *J. Comput. Chem*. **1992**, 13, 1011-1021.

21 Grossfield A. "WHAM: the weighted histogram analysis method", version 2.0.9, http://membrane.urmc.rochester.edu/wordpress/?page_id=126.

22 Barducci, A.; Bussi, G.; Parrinello, M. Well-Tempered Metadynamics: a smoothly converging and tunable free-energy method. *Phys. Rev. Lett.* **2008**, 100, 020603.
